# Quantitative Effect of Metal Artefact Reduction on CT-based attenuation correction in FDG PET/CT in patients with hip prosthesis

**DOI:** 10.1186/s40658-021-00414-2

**Published:** 2021-10-09

**Authors:** Maarten Haemels, Delphine Vandendriessche, Jeroen De Geeter, James Velghe, Maxence Vandekerckhove, Frank De Geeter

**Affiliations:** 1 Department of Nuclear Medicine, Algemeen Ziekenhuis Sint-Jan Brugge-Oostende, Ruddershove 10, 8000 Brugge, Belgium; 2 ELEC Department, Faculty of Applied Sciences, Vrije Universiteit Brussel, Building K - Room K.6.55/D2, Pleinlaan 2, 1050 Brussel, Belgium; 3 Nuclear Technology Center, UHasselt, Campus Diepenbeek, Agoralaan Building D, 3590 Diepenbeek, Belgium; 4 Department of Orthopedics, Algemeen Ziekenhuis Sint-Jan Brugge-Oostende, Ruddershove 10, 8000 Brugge, Belgium

**Keywords:** Metal artefact reduction, Hip prosthesis, Quantitative accuracy

## Abstract

**Background:**

Metal artefact reduction (MAR) techniques still are in limited use in positron emission tomography/computed tomography (PET/CT). This study aimed to investigate the effect of Smart MAR on quantitative PET analysis in the vicinity of hip prostheses.

**Materials and methods:**

Activities were measured on PET/CT images in 6 sources with tenfold activity concentration contrast to background, attached to the head, neck and the major trochanter of a human cadaveric femur, and in the same sources in similar locations after a hip prosthesis (titanium cup, ceramic head, chrome-cobalt stem) had been inserted into the femur. Measurements were compared between PET attenuation corrected using either conventional or MAR CT. In 38 patients harbouring 49 hip prostheses, standardized uptake values (SUV) in 6 periprosthetic regions and the bladder were compared between PET attenuation corrected with either conventional or MAR CT.

**Results:**

Using conventional CT, measured activity decreased with 2 to 13% when the prosthesis was inserted. Use of MAR CT increased measured activity by up to 11% compared with conventional CT and reduced the relative difference with the reference values to under 5% in all sources. In all regions, to the exception of the prosthesis shaft, SUV_mean_ increased significantly (*p* < 0.001) by use of MAR CT. Median (interquartile range) percentual increases of SUV_mean_ were 1.4 (0.0–4.2), 4.0 (1.8–7.8), 7.8 (4.1–12.4), 1.5 (0.0–3.2), 1.4 (0.8–2.8) in acetabulum, lateral neck, medial neck, lateral diaphysis and medial diaphysis, respectively. Except for the shaft, the coefficient of variation did not increase significantly. Except for the erratic changes in the prosthesis shaft, decreases in SUV_mean_ were rare and small. Bladder SUV_mean_ increased by 0.9% in patients with unilateral prosthesis and by 4.1% in patients with bilateral prosthesis.

**Conclusions:**

In a realistic hip prosthesis phantom, Smart MAR restores quantitative accuracy by recovering counts in underestimated sources. In patient studies, Smart MAR increases SUV in all areas surrounding the prosthesis, most markedly in the femoral neck region. This proves that underestimation of activity in the PET image is the most prevalent effect due to metal artefacts in the CT image in patients with hip prostheses. Smart MAR increases SUV in the urinary bladder, indicating effects at a distance from the prosthesis.

## Background

Metallic implants induce various artefacts in CT images. In PET/CT, these propagate into the PET reconstruction because the CT is used for attenuation correction [1–5]. Several approaches have been developed to mitigate metal artefacts [6]. Although these Metal Artefact Reduction (MAR) techniques have found widespread application in stand-alone CT, their use in PET/CT is still limited, in spite of evidence from phantom studies that they may improve quantitative accuracy [7–11]. Improved quantitative accuracy was also confirmed in 30 patients with a hip prosthesis [8] and in a further 16 patients with hip prosthesis belonging to a series of 28 oncological patients, although results in patients with hip prostheses were not reported separately from those with other metallic implants [11]. On the other hand, Nahmias et al., studying the effects of MAR in patients with dental implants, emphasized that the effects of MAR on PET quantification were less than the corresponding effects on CT [12]. This finding was corroborated in a population of miscellaneous metal implants [13]. Nevertheless, preliminary evidence has come from patient studies that MAR may be useful clinically [13, 14]. The number of patients with hip prosthesis included in these studies, however, has been limited: only 12 in [14] (of whom only 5 had ^18^F-FDG PET/CT) and 16 in [13].

In the present work, we sought to validate the commercially available software Smart MAR (GE Healthcare, Milwaukee, USA) [15] in patients with hip prostheses. To our knowledge, no studies have previously documented the effect of this software on PET/CT. We studied the effect of MAR on quantitative accuracy in a custom made phantom. We quantified the impact of MAR in patients with hip prostheses and mapped it topographically. This type of information was lacking, but may be important considering the potential role of ^18^F-FDG PET for diagnosis of periprosthetic joint infection [16–20].

## Methods

### Phantom experiment

**Fig. 1:**
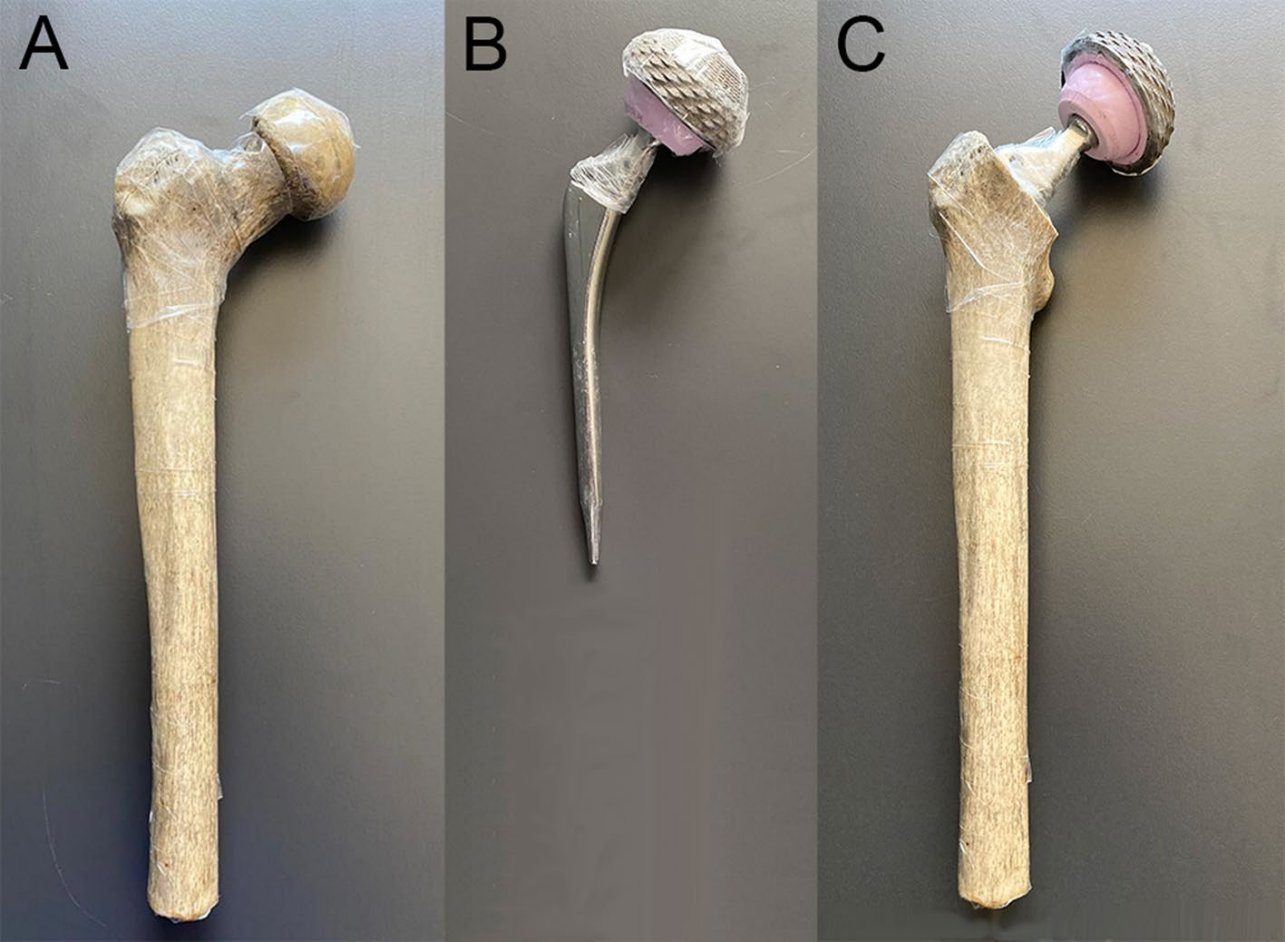
Femur and implant used in the phantom experiment. Panel **A** shows part of the femur, panel **B** the metallic implant, and panel **C** the assembly with the implant inserted in femur as used in the phantom experiment

**Fig. 2:**
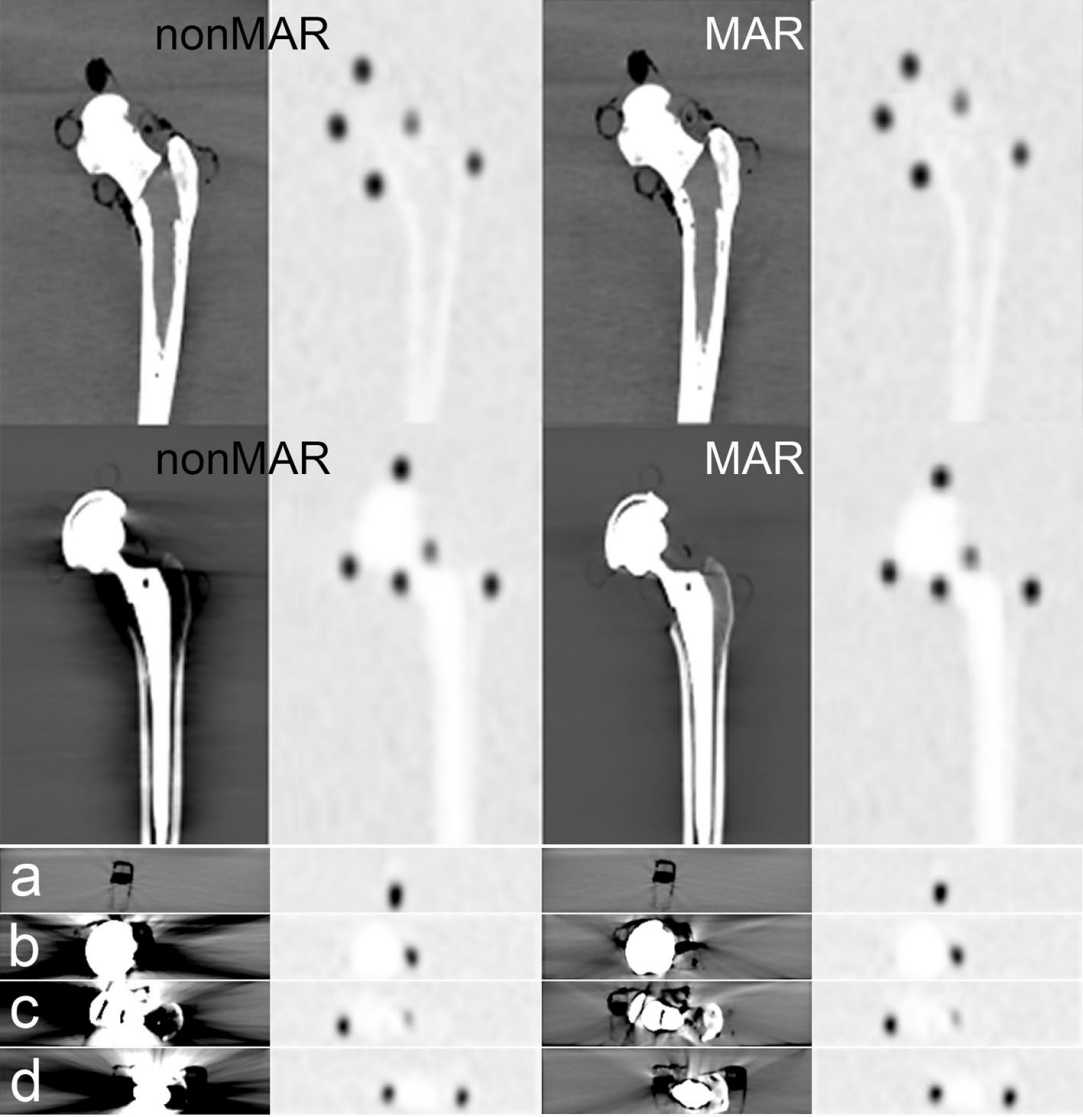
PET and CT images from the phantom experiment. Results of the scam experiment are shown in the upper row. Further rows show the results of the prosthesis experiment. The two columns to the left are not corrected by MAR, those to the right are. Corresponding CT (left) and PET (right) images are given. The top two rows show coronal images; to indicate the position of the sources, which were not all in the same coronal plane, the PET images are composites of several slices. The lower rows show axial slices at the level of the lateral acetabular source (row a), the lateral neck source (row b), the medial acetabular source (row c), and the medial neck source and the trochanteric source (row d). Some air visible in the sources is due to incomplete filling of the tubes. Note the dark streak artefacts around the prosthesis, which largely disappear from the MAR corrected CT. White streak artefacts are mainly situated anterior and posterior to the prosthesis

**Fig. 3:**
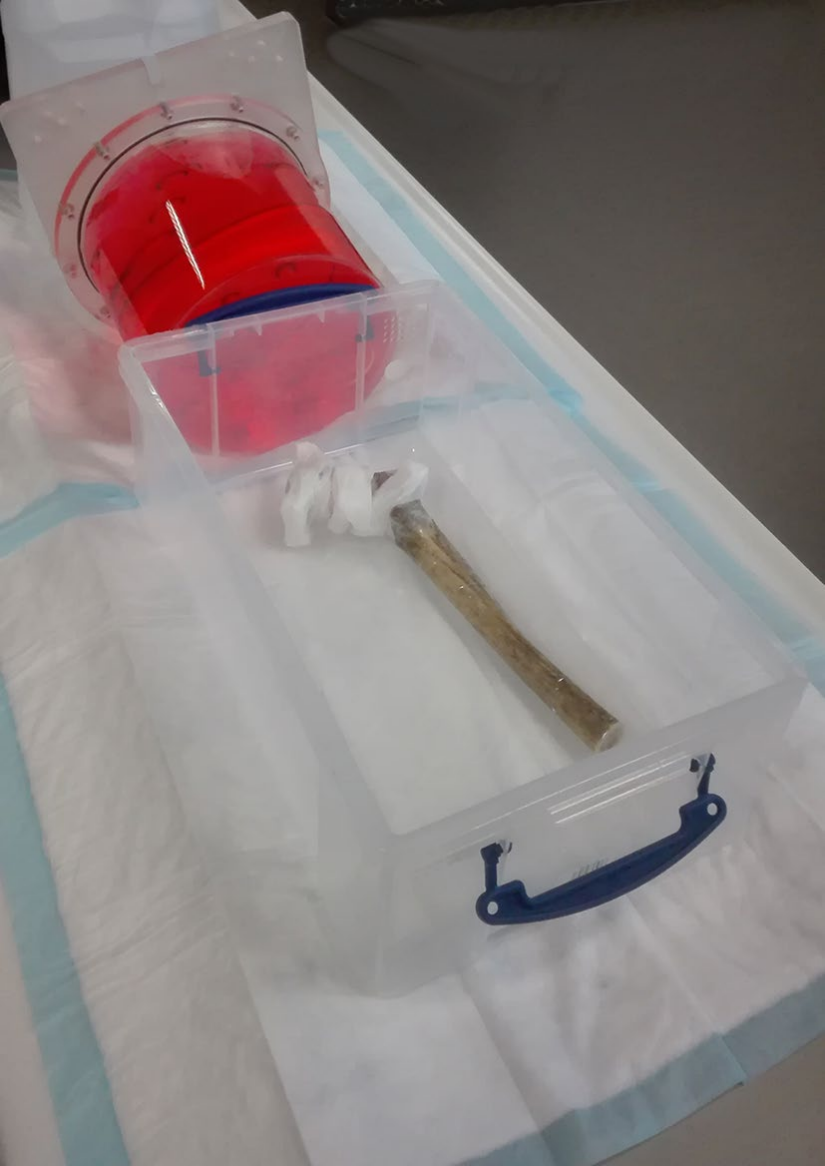
Experimental setup. The picture shows the femur-implant assembly in the container not yet filled with water. Note the tape securing the sources in place. The scatter phantom is placed in front

A human left femur was obtained from an anatomical lab. The distal third was sawn away to fit the remainder into a 42 by 24 by 15 cm polypropylene container. A neck osteotomy was performed, and the marrow cavity was reamed to fit the femoral stem of a prosthesis composed of a chrome-cobalt alloy (Fig. 1). The femur was covered in cellophane tape to ensure structural integrity. The titanium acetabular prosthesis component was taped over the ceramic joint in its correct anatomical position using Mefix surgical tape. 1.5 cc Eppendorf tubes (Eppendorf, Aarschot, Belgium) were filled with a solution of 2.975 MBq in 0.2 l water (or 14.88 MBq/l), so as to obtain an approximate target to background ratio of 10:1. They were fixed to the assembly by surgical tape in five positions: at the medial and lateral border of the cup, at the superolateral and inferomedial borders of the prosthesis neck and at the greater trochanter (Fig. 2). These locations were inspired by the Reinartz patterns of prosthetic infection [16, 17].The whole assembly was immersed in an aqueous solution of 7.5 MBq of ^18^F-FDG in slightly more than 5 L in the polypropylene container (the exact volume was unknown because some water had to be added to ensure complete immersion of the femur). A cylindrical acrylic uniformity phantom of 20 cm diameter and 18 cm height was positioned in front of the plastic container. It was filled with a solution of 8.31 MBq in 5.701 l, so as to simulate abdominal/pelvic background. Figure 3 shows the experimental setup.

Image acquisition was performed in the same PET/CT scanner as used for clinical imaging, and the scanning parameters both for PET and CT were identical (see below).

Next, the prosthesis was extracted from the femur, the original femur head was taped to the diaphysis and the Eppendorf tubes were each taped to the femur in similar positions as for the first experiment, although we had to take into account the larger dimension of the anatomical neck as opposed to the prosthesis neck and the smaller surface area of the femoral head. To keep the femur immersed, it was taped to the bottom of the container. An acquisition with identical parameters as the first one was subsequently performed.

Image reconstruction was the same as for patient data (see below).

On the PET images corrected for attenuation by MAR CT, volumes of interests (VOIs) were positioned on the 5 radioactive sources surrounding the femur using autocontour software with a relative threshold of 42% (Volume Viewer, GE Healthcare, Milwaukee, USA). These were then cloned to the PET attenuation corrected by the CT without MAR. In each VOI, the mean and maximum activity (kBq/ml) were measured. This was repeated twice: for the PET data with and without the total hip prosthesis inserted into the phantom. Activities on the second acquisition were corrected for radioactive decay between the acquisitions.

### Patients

Thirty-eight 2‐[^18^F]fluoro‐2‐deoxy‐D‐glucose (^18^F-FDG) positron emission tomography/computed tomography (PET/CT) scans were retrospectively selected from all clinically indicated whole-body ^18^F-FDG PET/CT scans performed at the department of nuclear medicine in AZ Sint-Jan Bruges (Belgium) between October 30, 2018, and July 05, 2019, on basis of the following criteria: 1. presence of at least one total hip prosthesis. Resurfacing prostheses, short stem prostheses and hip screws were excluded from the study; 2. absence of registration artefacts due to patient movement between PET and CT; and 3. availability of correctly saved raw PET data and CT reconstructions with and without MAR. When a patient underwent multiple ^18^F-FDG PET/CT scans during the inclusion period, only the first one was taken into account.

Most of the patients (= 35) selected underwent PET for staging or follow-up of malignancy. In one patient, a periprosthetic hip infection was suspected. In another patient, PET was performed to elucidate a lung consolidation; a third patient had nonspecific constitutional symptoms. Eleven patients had bilateral hip prostheses, for a total of 49 hip prostheses analysed. Patients were between 60 and 89 years old (mean 73, standard deviation 7.6 year); their BMI ranged from 17.7 up to 39.3 (mean 25.7, standard deviation 4.7).

### PET/CT imaging

All PET/CT examinations were performed on a Discovery MI 15 cm axial field-of-view PET/CT camera (GE Healthcare, Milwaukee, USA) [21]. Patients fasted for at least 6 h before ^18^F-FDG injection and had blood glucose confirmed to be below 200 mg/dl before injection. The amount of tracer administered was based on the body mass index of the patient (BMI < 20: 1.5 MBq/kg; BMI 20–26.5: 2 MBq/kg; BMI > 26,5: 2.5 MBq/kg). ^18^F-FDG was injected intravenously under standard conditions. Imaging was started after rest for 60 min in a comfortable position. Patients were positioned in the scanner with their arms raised.

PET consisted of 7 to 9 bed positions of 2 min duration each, from the skull vertex to the mid-thigh. Reconstruction used a three-dimensional ordered subset expectation maximization (OSEM) algorithm (4 iterations, 8 subsets, Gaussian post-filtering 6.0 mm full width at half maximum, heavy Z-axis filter, matrix size 256 × 256, slice thickness 2.5 mm) with time-of-flight and point spread function correction (VPFX -S, GE Healthcare, Milwaukee, USA).

CT used a tube voltage of 120 keV and Smart mA automatic exposure control (GE Healthcare, Milwaukee). Intravenous contrast (Xenetix 350, Guerbet, France) was used depending on the clinical indication. For metal artefact reduction, the GE Smart MAR algorithm was used [15]. CT reconstructions were made with and without MAR. PET data were attenuation corrected using these MAR and nonMAR reconstructed CT data.

### Image analysis

**Fig. 4:**
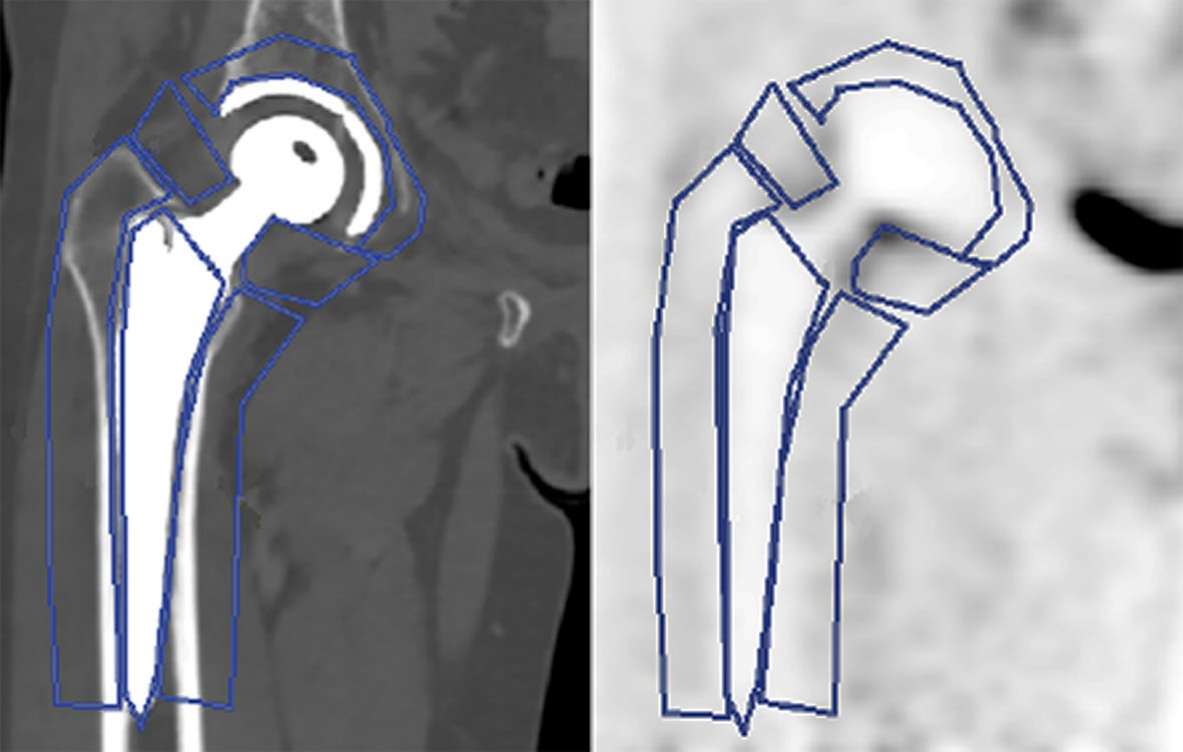
Typical ROIs outlined on corresponding CT and PET images. ROIs correspond to acetabular, medial and lateral neck, medial and lateral diaphysis, and shaft areas. They were drawn in coronal planes through the midplane of the prosthesis

Analysis of PET images was performed using the Volume Viewer software on the Advantage Workstation (GE Healthcare, Milwaukee, USA). PET and CT datasets were spatially registered and reoriented so that the coronal plane corresponded as good as possible to the midplane of the prosthesis. Two-dimensional regions of interests (ROIs) were drawn manually in six locations around the prosthesis on the MAR corrected CT images: on the acetabulum, medially and laterally in the neck region, on the prosthesis shaft, and medially and laterally on the femoral diaphysis. The shaft of the prosthesis served as a control (Fig. 4). Afterwards, these regions were copied to the other datasets. In each ROI, mean and maximum standardized uptake values (SUV_mean_ and SUV_max_) (g/ml) as well as the standard deviation of the SUV were measured. The SUV was calculated as the activity concentration in the PET image divided by the injected activity per g of body weight. A three-dimensional volume of interest was drawn over the urinary bladder using the autocontour software with a relative threshold of 42%.

### Statistical analysis

Two-way repeated measures ANOVA was performed to compare SUV_mean_ in the various ROIs surrounding the prosthesis in images reconstructed using MAR or without using MAR. Greenhouse–Geisser correction was applied in case of violation of the sphericity assumption. Significant ANOVA-testing was followed by pairwise comparisons between groups using *t* tests with Bonferroni adjustment.

In each region, the relative change of SUV_mean_ on using MAR-corrected CT for PET reconstruction was calculated as (SUV_mean_ in MAR-PET − SUV_mean_ in nonMAR-PET)/(SUV_mean_ in nonMAR-PET). The sign test was used to test in each ROI whether the median relative change was significant. The lower limit of the one-sided confidence interval based on the sign test was reported. Differences of the relative change between regions were tested by robust ANOVA with 20% trimmed means and 2000 bootstrap samples. Robust ANOVA was followed by robust post hoc tests using 20% trimmed means and 5000 bootstrap samples and corrected for the number of tests. All testing was performed two-sided.

Correlation plots between SUV_mean_ in MAR and nonMAR PETs were constructed using the data from all regions in all patients. Bland–Altman plots were constructed using log-transformed data, because the differences between MAR and nonMAR SUV_mean_ increased with increasing SUV_mean_.

Similar analyses were performed on SUV_max_ and on the coefficient of variation of the SUV.

In 37 patients, bladder volume and SUV data were compared between PET reconstructions with MAR-corrected CTs and those with uncorrected CTs. One patient was excluded from this analysis, because no substantial bladder activity was present owing to an indwelling catheter. The sign test was used for the comparison.

The relative changes of the bladder parameters on using MAR-CT versus conventional CT for PET attenuation correction were calculated. The sign test was used to test whether these were significant. Wilcoxon’s rank sum test was used to compare the relative changes between patients with unilateral and bilateral prostheses.

Significance was called when *p* was less than 0.05.

All statistical testing and graphics was performed in R version 4.0.1 [22], and figures were produced using the package ggplot2 [23]. Robust statistical tests were performed by the WRS (Wilcox’ Robust Statistics) package [24].

## Results

### Phantom experiment

**Fig. 5:**
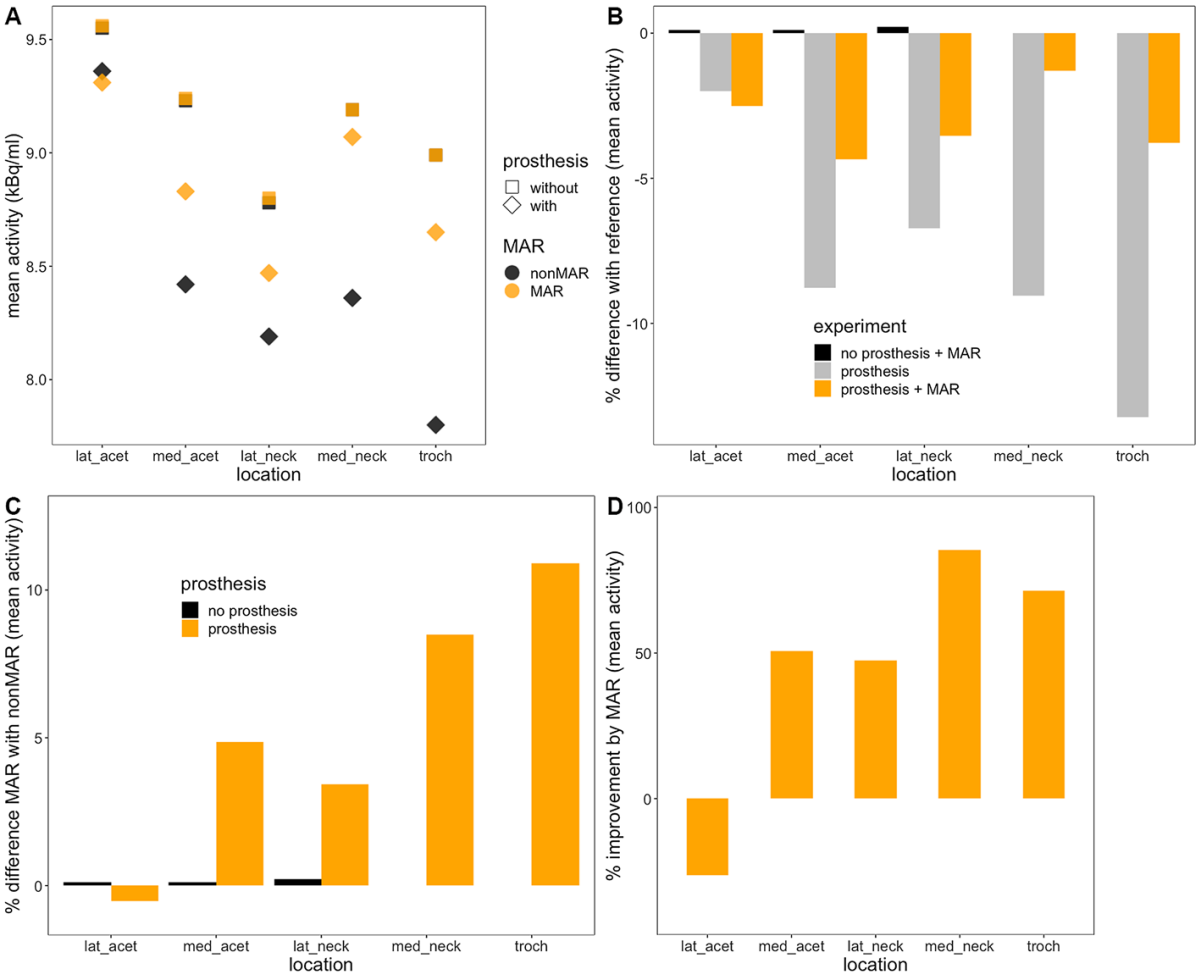
Mean activity in the phantom experiment. Panel **A** depicts the mean activity measured in the 5 sources in the scam and prosthesis experiments using MAR CT (orange symbols) or conventional CT (black symbols) for attenuation correction. Panel **B** gives the percentual differences between the measurements and the reference value (in the scam experiment without MAR), panel **C** the differences between MAR and nonMAR PET. Panel **D** indicates the percentage of the error in the prosthesis experiment that is recovered by MAR. *lat_acet* lateral acetabular source; *med_acet* medial acetabular source; *lat_neck* lateral neck source; *med_neck* medial neck source; *troch* trochanteric source

**Fig. 6:**
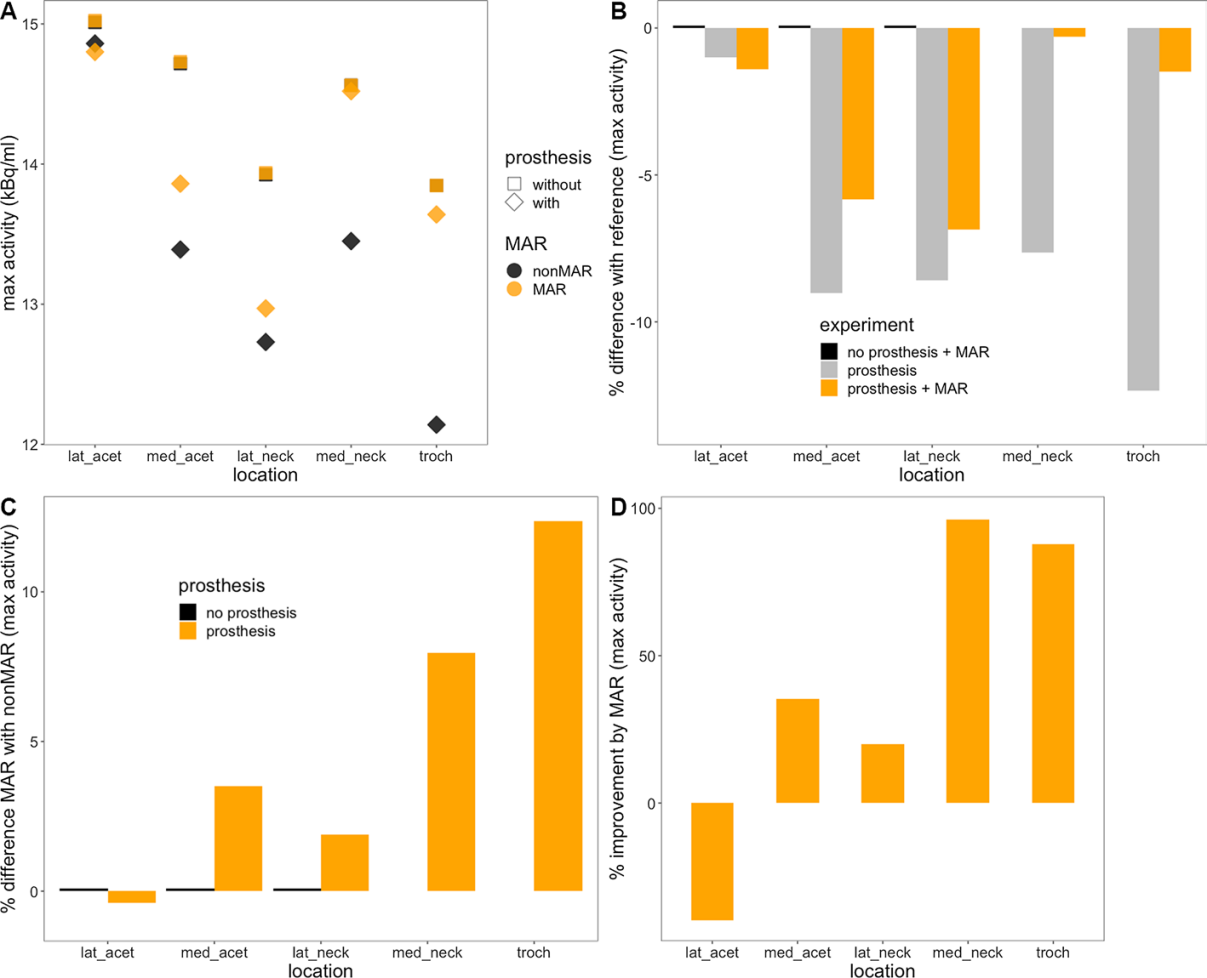
Maximum activity in the phantom experiment. Panel **A** depicts the maximum activity measured in the 5 sources in the scam and prosthesis experiments using MAR CT (orange symbols) or conventional CT (black symbols) for attenuation correction. Panel **B** gives the percentual differences between the measurements and the reference value (in the scam experiment without MAR), panel **C** the differences between MAR and nonMAR PET. Panel **D** indicates the percentage of the error in the prosthesis experiment that is recovered by MAR. *lat_acet* lateral acetabular source; *med_acet* medial acetabular source; *lat_neck* lateral neck source; *med_neck* medial neck source; *troch* trochanteric source

The results from the phantom experiment are depicted in Fig. 5. True activity (14.88 MBq/l) was not recovered because of the partial volume effect; activity measurements in the scam experiment (using only the femur without the prosthesis) were used as a reference. As expected, MAR only had very little or no effect in the scam experiment. With the prosthesis inserted, less activity was measured in all of the target sources. The difference between measured and reference activity was highest in the source at the greater trochanter (13%), but was also important medially in the neck (9%), medially at the acetabulum (9%) and laterally in the neck (7%); at the lateral acetabulum, it was only 2% (panel B). MAR improved these measurements quite well, increasing the measured activity by 3 to 11 (panel C) and reducing the relative difference with the reference to under 5% (panel B). The percentage of underestimation that was recovered by the use of MAR varied from 47% in the lateral neck to over 85% in the medial neck (panel D). In the lateral acetabular source, which was the one closest to the reference activity in the prosthesis experiment (at about − 2%), MAR increased the difference slightly to − 2.5% of the reference activity. On inspection of the combined PET and CT images, this source was the only one which did not lie in a dark streak artefact. Analysis of the maximum activity in the sources showed qualitatively similar results (Fig. 6).

### Patient data

#### SUV_mean_

**Fig. 7:**
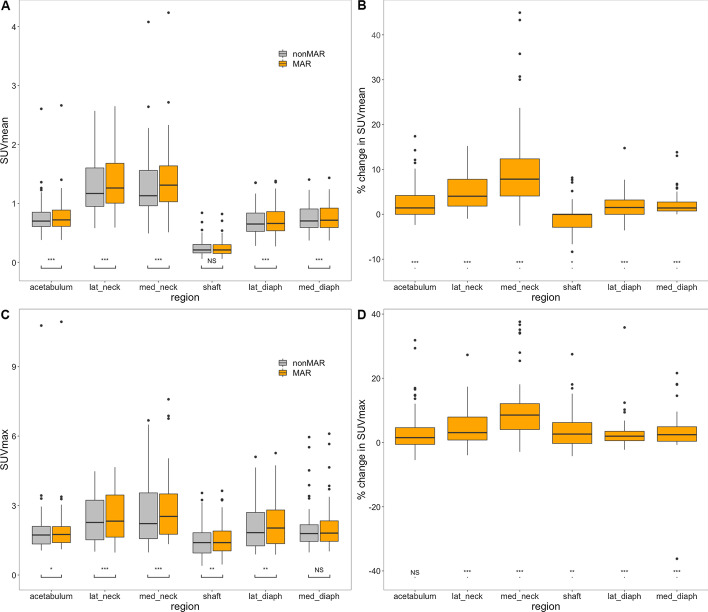
Effect of MAR on SUV_mean_ and SUV_max_ in regions around the prostheses. Panels **A** and **C** are boxplots describing the SUV readings according to whether or not MAR was applied for PET attenuation correction. Panels **B** and **D** are boxplots describing the percentual change of SUV induced by MAR. Boxes represent the interquartile range, and the horizontal line represents the median. Whiskers extend to the smallest, respectively, largest values at most 1.5*the interquartile range from the 25th and 75th percentiles. Dots represent outliers. Statistical significance of the difference with use of MAR is indicated in panels **A** and **C**; in panels **B** and **D** it is indicated whether the percentual increase in SUV differs significantly from 0 (NS denotes not significant, *p < 0.05, **p < 0.01, ***p < 0.001). *lat_neck* lateral neck; *med_neck* medial neck; *lat_diaph* lateral diaphysis; *med_diaph* medial diaphysis

Repeated measures ANOVA showed that SUV_mean_ depended both on the area it was measured in (Greenhouse–Geisser corrected *p* < 0.001) and on the use of MAR-CT for attenuation correction of the PET (*p* < 0.001), and that these interacted significantly (Greenhouse–Geisser corrected *p* < 0.001) (Fig. 7 panel A). As expected, SUV_mean_ in the shaft ROI was significantly lower than in all other regions (Bonferroni corrected *p* < 0.001 both with and without MAR). SUV_mean_ was higher in the neck regions than in all others (*p* < 0.001 both without and with MAR). MAR had no significant effect in the shaft region. In all other regions, MAR increased SUV_mean_ significantly (*p* < 0.001 for all regions).

**Fig. 8:**
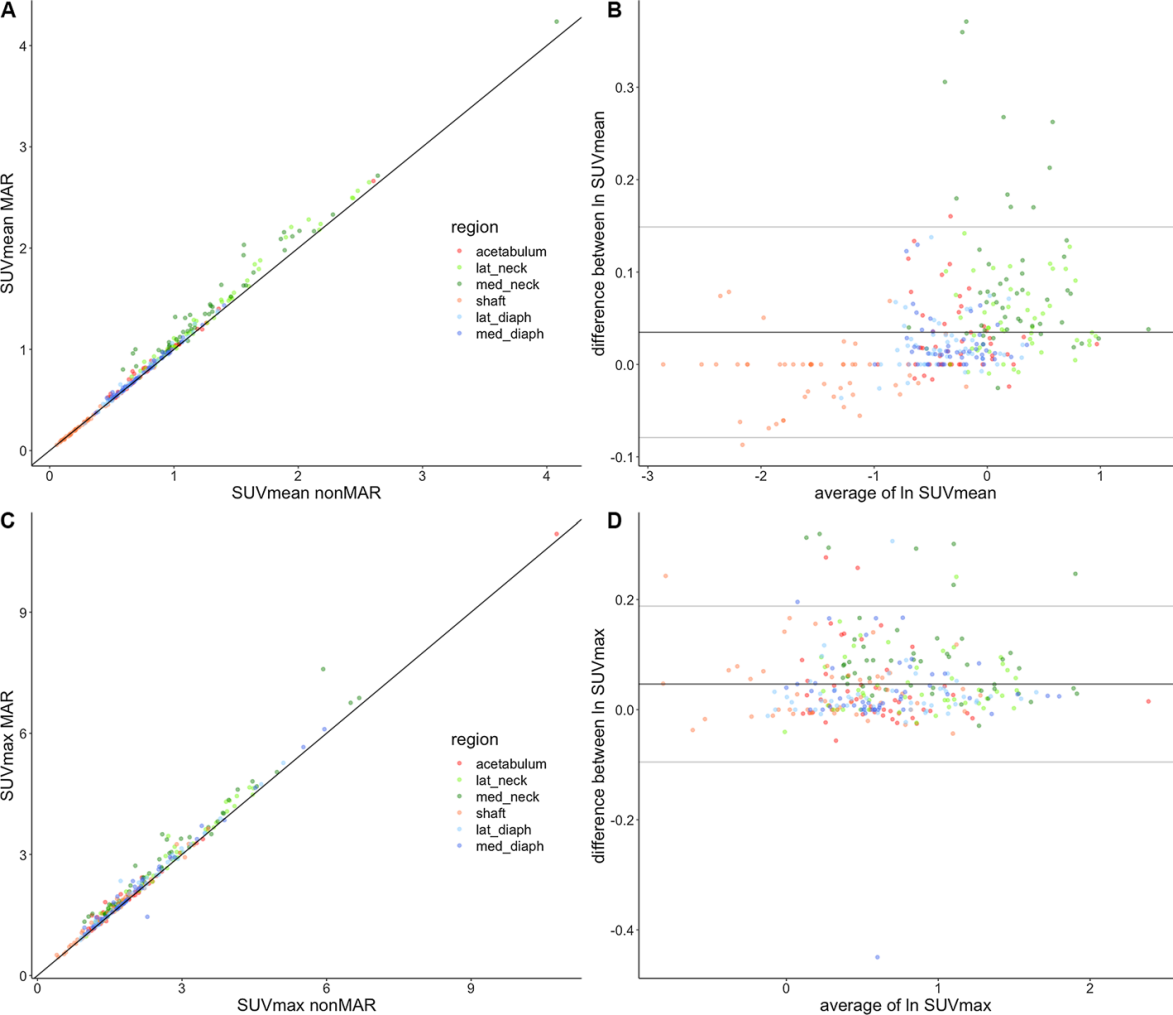
Correlation and Bland–Altman plots of SUV with and without MAR in regions around the prostheses. In the correlation plots (panels **A**, SUV_mean_ and **C**, SUV_max_), almost all points lie above the line of identity. The Bland–Altman plots (panels **B**, SUV_mean_ and **D**, SUV_max_) use log-transformed data, and horizontal lines indicate the mean value and 95% confidence boundaries of the differences. Data from different regions are colour coded. *lat_neck* lateral neck; *med_neck* medial neck; *lat_diaphysis* lateral diaphysis; *med_diaphysis* medial diaphysis

Percentual changes of SUV_mean_ by using MAR-corrected CT for PET reconstruction are illustrated in Fig. 7, panel B and further described in Table 1. In all regions, except for the shaft, these were significantly different from 0 (*p* < 0.001) and positive; in the shaft, they were also significantly different from 0 (*p* < 0.05), but negative. In all regions, again except for the shaft, SUV_mean_ increased in PET images reconstructed with MAR-corrected CT images in the overwhelming majority of patients. As can be seen in Fig. 8, panels A and B, and in Table 2, decreases in SUV_mean_ with MAR were always limited and did only occur in areas with already low SUV_mean_ on nonMAR PET, in particular in the shaft region. Robust ANOVA on the relative SUV_mean_ changes showed highly significant differences between regions (*p* < 0.001). Robust post hoc tests showed relative changes in SUV_mean_ to be significantly lower in the shaft than in all other ROIs (Fig. 7, panel B). Relative SUV_mean_ changes were significantly higher in the medial neck ROI than in the acetabular and diaphyseal ROIs and significantly higher in the lateral neck ROI than in both diaphyseal ROIs.

**Table 1:** Quantiles of % change of SUV_mean_ around prostheses by MAR CT

Region	Acetabulum	Lat_neck	Med_neck	Shaft	Lat_diaph	Med_diaph
Minimum	− 2.36	− 0.98	− 2.52	− 8.33	− 3.57	0.00
5% quantile	− 1.39	− 0.72	2.23	− 6.16	− 1.54	0.00
25% quantile	0.00	1.83	4.08	− 2.88	0.00	0.75
50% quantile	1.43	4.04	7.84	0.00	1.53	1.42
75% quantile	4.21	7.81	12.37	0.00	3.20	2.75
95% quantile	11.86	11.39	33.73	6.34	7.21	6.70
Maximum	17.39	15.24	44.93	8.16	14.76	13.83
2-sided p§	***	***	***	*†	***	***
Lower limit¶	0.65	3.13	5.11	− 1.82	1.14	1.13

The table gives the quantiles of the percentual change of SUV_mean_ (in g/ml) in various regions around the prostheses induced by the use of MAR CT for PET attenuation correction

*lat_neck* lateral neck region; *med_neck* medial neck region; *lat_diaph* lateral diaphysis region; *med_diaph* medial diaphysis region)

^§^on sign test. * = *p* < 0.05; ** = *p* < 0.01; *** = *p* < 0.001

^†^and lower than 0, whereas in all other regions the median was higher than 0

^¶^ of one-sided 95% confidence interval

**Table 2:** Change of SUV_mean_ induced by MAR in regions around the prosthesis

Region	Decrease	No change	Increase
Acetabulum	4	14	31
Lat_neck	4	2	43
Med_neck	1	0	48
Shaft	20	22	7
Lat_diaphysis	5	8	36
Med_diaphysis	0	10	39

The numbers correspond to the numbers of patients in whom a given change occurs in a given region

*lat_neck* lateral neck region; *med_neck* medial neck region; *lat_diaphysis* lateral diaphysis region; *med_diaphysis* medial diaphysis region

#### SUV_max_

SUV_max_ was affected by the region it was measured in (Greenhouse–Geisser corrected *p* < 0.001) as well as by the use of MAR (*p* < 0.001) and by their interaction (Greenhouse–Geisser corrected *p* < 0.001) (Fig. 7, panel C). It was smaller in the shaft than in all other regions, both without and with MAR (*p* < 0.001 for all, except *p* < 0.01 for medial diaphysis with MAR, and not significant for acetabulum without and with MAR). SUV_max_ was higher in the lateral neck than in the lateral diaphysis (*p* < 0.05 without MAR, *p* < 0.01 with MAR), in the medial neck than in the lateral diaphysis (*p* < 0.01 with MAR), in the medial neck than in the medial diaphysis (*p* < 0.05 without MAR, *p* < 0.01 with MAR) and in the medial neck than in the acetabulum (*p* < 0.05 with MAR). Significant increases were accomplished by using MAR CT for PET attenuation correction in all regions, except the medial diaphysis (*p* < 0.05 in acetabulum, *p* < 0.01 in shaft and lateral diaphysis, *p* < 0.001 in neck regions).

Relative changes of SUV_max_ induced by MAR were significant in all regions except the acetabulum (*p* < 0.01 in shaft, *p* < 0.001 elsewhere) (Fig. 7, panel D). In all regions, SUV_max_ increased with MAR in the overwhelming majority of patients (Fig. 8, panels C and D). Robust ANOVA on the relative change of SUV_max_ showed highly significant differences between regions (*p* < 0.001) (Fig. 7, panel D). Post hoc testing showed that the relative change of SUV_max_ was higher in the medial neck region than in all other regions.

#### Coefficient of variation

**Fig. 9:**
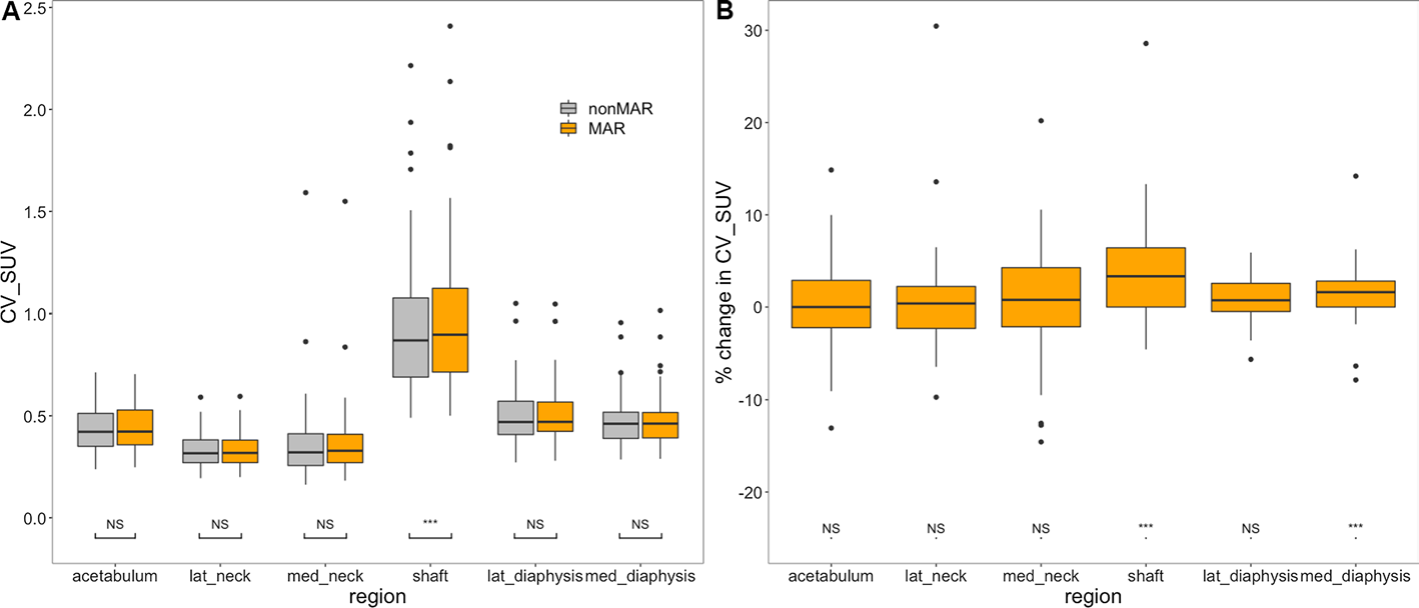
Effect of MAR on variation coefficient of the SUV in regions around the prostheses. Panel **A** is a boxplot describing the variation coefficient of the SUV according to whether or not MAR was applied for PET attenuation correction. Panel **B** is a boxplot describing the percentual change of the variation coefficient induced by MAR. Boxes and whiskers are as in Fig. 7. Statistical significance of the difference with use of MAR is indicated in panel **A**; in panel **B** it is indicated whether the percentual increase in the variation coefficient differs significantly from 0 (NS denotes not significant, *p < 0.05, **p < 0.01, ***p < 0.001. *lat_neck* lateral neck; *med_neck* medial neck; *lat_diaph* lateral diaphysis; *med_diaph* medial diaphysis; *CV_SUV* variation coefficient of SUV

Repeated measures ANOVA showed that the coefficient of variation within regions was affected by both the region (Greenhouse–Geisser corrected *p* < 0.001) and the use of MAR CT for attenuation correction of the PET (*p* < 0.001), and that these interacted significantly (Greenhouse–Geisser corrected *p* < 0.001). This is illustrated in Fig. 9, panel A. Variation was larger in the shaft than in all other regions (Bonferroni corrected *p* < 0.001, both on MAR and nonMAR PET) and was lower in the lateral neck region than in the diaphyseal regions and the acetabulum (*p* < 0.001, both on MAR and nonMAR PET). Only in the shaft region, MAR increased variability significantly (*p* < 0.001).

Robust ANOVA showed significant differences of the relative change of variation coefficients between regions (*p* < 0.01) and robust post hoc testing located these between the shaft and the acetabulum, the lateral neck and the lateral diaphyseal region (Fig. 9, panel B). Indeed, only in the shaft (*p* < 0.001) and medial diaphysis (*p* < 0.001), the relative increase in the variation coefficient upon use of MAR was significant.

#### Bladder

**Fig. 10:**
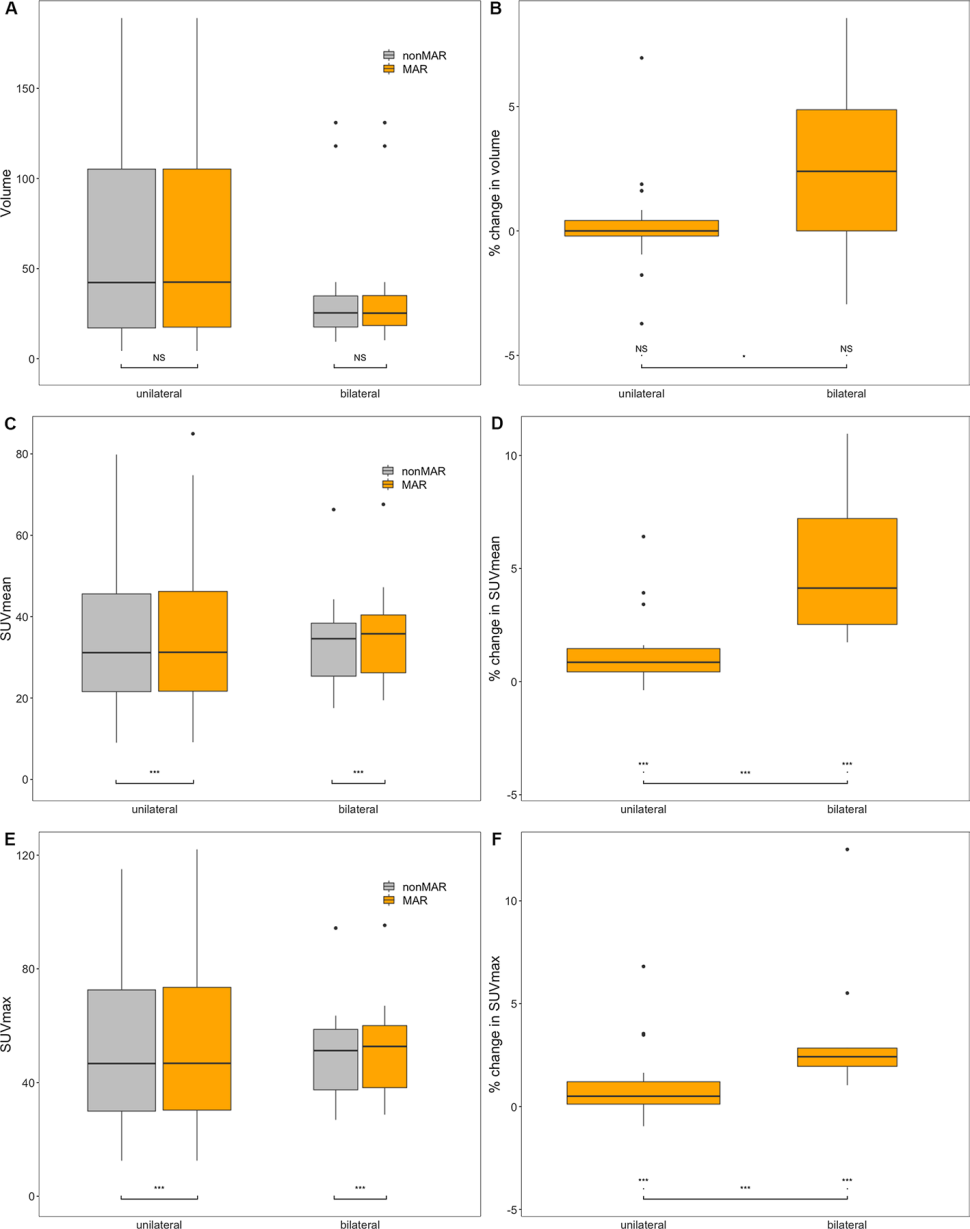
Bladder measurements. Panels **A**, **C** and **E** are boxplots describing the volume, SUV_mean_ and SUV_max_ of the bladder, according to the presence of unilateral or bilateral hip prostheses and to the use of MAR CT for attenuation correction. Panels **B**, **D** and **F** are boxplots describing the percentual changes of the measured volume, SUV_mean_ and SUV_max_ induced by MAR. Boxes and whiskers are as in Fig. 7. The width of the boxes is proportional to the square root of the number of observations. Statistical significance of the difference with use of MAR is indicated in panels **A**, **C** and **E**; in panels **B**, **D** and **F** it is indicated whether the percentual increase in SUV differs significantly from 0 (NS denotes not significant, *p < 0.05, **p < 0.01, ***p < 0.001)

The bladder volume measured was not significantly affected by the use of MAR-CT for PET reconstruction (Fig. 10, panel A). To the contrary, SUV_mean_ was significantly increased (*p* < 0.001) when MAR-CT was used, in all patients taken together and in those with unilateral or bilateral prosthesis taken apart (Fig. 10, panel C). This held also for SUV_max_ (Fig. 10, panel E).

**Fig. 11:**
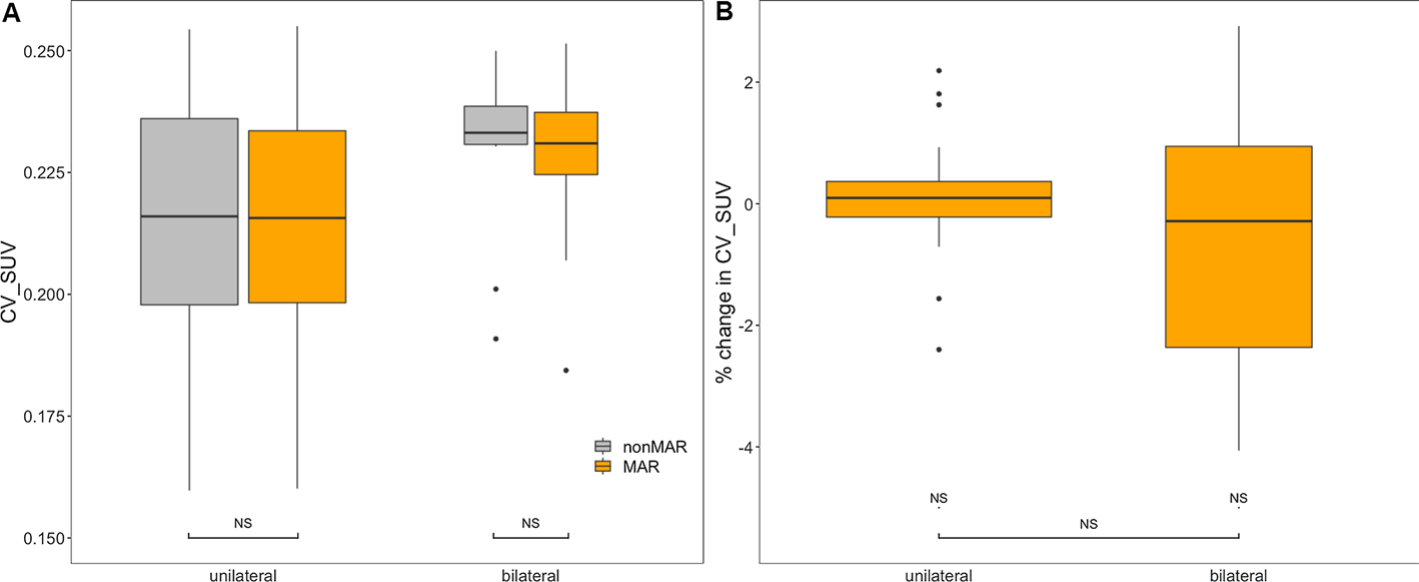
Effect of MAR on the variation coefficient of the SUV in the bladder. Panel **A** is a boxplot describing the variation coefficient of the SUV according to whether or not MAR was applied for PET attenuation correction. Panel **B** is a boxplot describing the percentual change of the variation coefficient induced by MAR. Boxes and whiskers are as in Fig. 7. Statistical significance of the difference with use of MAR is indicated in panel **A**; in panel **B** it is indicated whether the percentual increase in the variation coefficient differs significantly from 0 (NS denotes not significant, *p < 0.05, **p < 0.01, ***p < 0.001). *CV_SUV* variation coefficient of SUV

The relative change in measured volume with MAR was not significant in all patients combined, neither in patients with bilateral or unilateral prosthesis taken separately, although it was significantly higher in patients with bilateral prosthesis than in those with unilateral prosthesis (Fig. 10, panel B). On the other hand, relative increases in SUV_mean_ and SUV_max_ in the bladder were significant both in all patients combined and in those with unilateral or bilateral prostheses taken apart (*p* < 0.001) (Fig. 10, panels D and F). They were higher in patients with bilateral prosthesis than in those with unilateral prosthesis (*p* < 0.001 for SUV_mean_ and *p* < 0.001 for SUV_max_). SUV_mean_ increased by 0.9% (interquartile range (IQR) from 0.4 to 1.5%) in patients with unilateral prosthesis and by 4.1% (IQR from 2.5 to 7.2%) in patients with bilateral prosthesis. SUV_max_ increased by 0.5% (IQR from 0.1 to 1.2%) in patients with unilateral prosthesis and by 2.4% (interquartile range from 2.0 to 2.8%) in patients with bilateral prosthesis. The coefficient of variation in the bladder did not change significantly by the use of MAR (Fig. 11).

## Discussion

The results of our phantom experiment (Fig. 5) indicate that the use of MAR-CT for attenuation correction of PET enhances quantitative accuracy. It reduced the relative errors on the measurements to less than 5% of the reference activity. Our results confirm the better quantitative accuracy obtained with MAR in several phantom studies with hip replacements [7, 9, 11], a cardiac pacemaker [8], or dental metalwork [10]. The relative recovery by MAR of underestimated activity in our experiment was similar to that reported in underestimated areas [8, 11]. Some of the previous studies used custom made software [7–9], whereas others used iMAR [10, 11], which is available commercially; to our knowledge, no results have been published with Smart MAR. Like Harnish et al. [9], we determined the reference activity in a scam experiment in which metal was absent, thereby avoiding partial volume effects which would have been introduced by comparing measured activity in small sources to the actual activity in them. Other authors used target to background ratios in sources in positions not affected by CT artefacts as a reference [7], or just measured background activity in areas not affected by metal artefacts on CT as a reference [8, 10, 11]. The strength of our experimental design further resides in a rather physiological setup using human bone and several rather small target sources. Limitations include absence of bony and soft tissue structures in the pelvis and slight differences in the disposition of the sources between the scam and real experiment, due to geometric differences between the prosthesis and the actual femur neck and head.

In all sources, except for the lateral acetabular one, measured activity increased with MAR, depending on the position of the source. The slight decrease in activity in the source at the lateral acetabular border—the only source not surrounded by dark streak artefacts—may be explained as a statistical fluctuation. Previous phantom experiments have shown either overestimation only [3, 9] or both over- and underestimation [4, 7, 8, 10] of activity measurements on PET induced by CT artefacts. Flare artefacts (bright streak artefacts corresponding to overestimation of true attenuation) and dark streak artefacts may coexist on CT, and the effect of MAR is predicted to be contingent on the precise area where it is measured. For example, in the experiment described in [9], the figures indicate that the source was positioned in a flare artefact, and accordingly, its activity was overestimated.

In patients, the use of MAR CT for attenuation correction of PET slightly decreased SUV_mean_ in the shaft region, which is largely composed of metal. SUV_max_ increased somewhat in this region, as could be expected from an extremum parameter. Although variability was already higher in the shaft than in any other region, the shaft was the only region where MAR increased it further, reflecting disparate effects in different areas within the region. At the level of the region as a whole, disparate effects among patients were observed as well; this was the only region in which MAR either induced slight decreases or did not change SUV_mean_ in a substantial number of patients.

In all other regions, MAR brought about significant increases in both SUV_mean_ and SUV_max_, their median ranging from 1.4 to 7.8% and from 2.0 to 8.6%, respectively (Figs. 7 and 8). The largest increases were observed in the neck regions, as expected from the mass of metal present in the neighbourhood of these regions (Fig. 1). As in our phantom experiment, they were larger in the medial neck region than in the lateral. Sizeable effects were also found in the diaphyseal and acetabular regions, again in accordance with our phantom experiment. The acetabular region averages the results for sources in the medial and lateral acetabulum. In our phantom experiment, the effect of MAR was at its largest for the source at the trochanter, but no isolated ROI representing this area was used for analysis of patient data. In the large lateral diaphysis region that we used for patient data, the effect may have been diluted. The significant increases in SUV that we found may seem to be in contrast with earlier reports which described little influence on PET images [12, 13]. However, one study only dealt with dental metalwork [12] and the other included only 16 patients with hip endoprostheses and did not measure activity in the bone surrounding the metal [13].

**Fig. 12:**
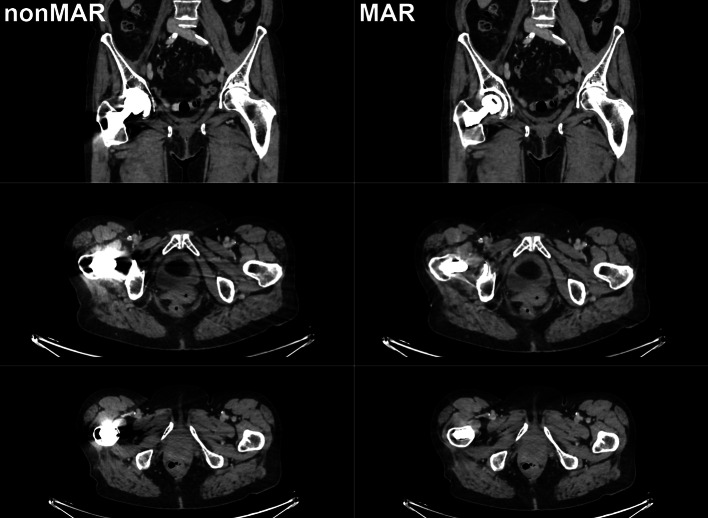
CT images with and without MAR in a patient with unilateral hip prosthesis. Selected coronal (upper row) and axial (second and third row) slices are shown from CT reconstructed without MAR (left column) and CT reconstructed with MAR (right column)

**Fig. 13:**
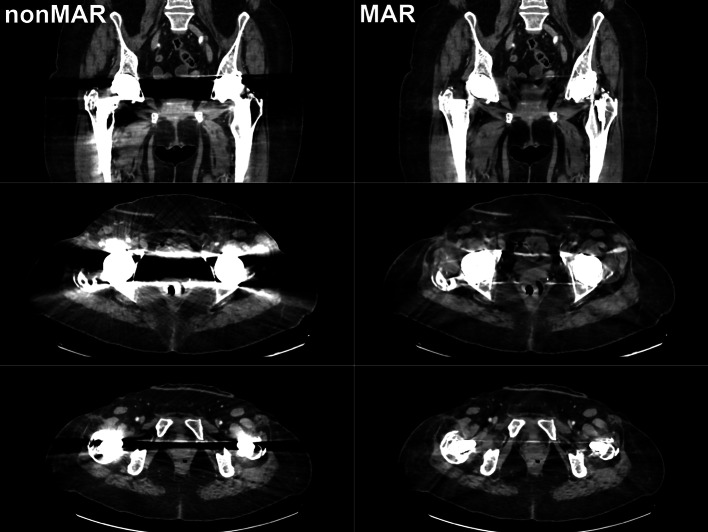
CT images with and without MAR in a patient with bilateral hip prostheses. Selected coronal (upper row) and axial (second and third row) slices are shown from CT reconstructed without MAR (left column) and CT reconstructed with MAR (right column)

That the predominant effect of MAR was to increase SUV in the areas surrounding the prostheses, does not contradict the possibility of overestimation of activity in particular areas around the prosthesis, such as have been reported clinically [5, 8, 11, 14]. However, overestimation seems confined to small regions and would typically only be picked up by small (10 to 15 mm diameter) regions of interest as used in [8, 11, 14] and specifically directed to bright streak artefacts. Both in our phantom experiment and in patients, bright streak artefacts indeed did occur, but were mainly oriented in the anteroposterior direction (Figs. 2, 12 and 13). In the larger midplane regions that we used, these would not be expected to affect our measurements to a large extent, and overestimation it is prevailed upon by underestimation.

The somewhat larger variability of the SUV in the diaphyseal regions that we observed, as reflected by a higher coefficient of variation, can easily be explained by the larger area of these regions. It is of more importance that, except for the shaft and medial diaphysis region, variability did not significantly increase by using MAR CT for attenuation correction (Fig. 9). This indicates that the effect of MAR is homogeneous in most regions.

Our results indicate that while the use of Smart MAR does not affect the autocontoured bladder volume, it increases bladder SUV_mean_ and SUV_max,_ without increasing variability of the bladder SUV (Figs. 10 and 11). Not unexpectedly, the effect is even larger in patients with bilateral prosthesis, in whom it is on the order of 4%, whereas in patients with unilateral prosthesis it is on the order of 1%. This effect of MAR stands in contrast to the result obtained by Reinert et al. [13], who found that iterative Metal Artefact Reduction (iMAR) did not significantly change muscular or bladder SUVs in the vicinity of a hip prosthesis. Several factors may explain this discrepancy. First, the software for the MAR used by these authors differed from ours. Second, we used the autocontoured bladder volume, whereas Reinert et al. [13] used a circular ROI. Although they choose a ROI ‘as large as possible in the structure of interest’, autocontouring the bladder is more likely to include peripheral bladder areas than is enforcing a circular ROI on the bladder. Precisely these peripheral regions may harbour more important effects of MAR. Third, they only included 16 patients with a hip prosthesis.

Although bladder activity is of little importance for clinical PET, our findings testify to the influence of metal artefacts on the pelvic soft tissue reading of PET. This was also documented by the phantom experiment and clinical examples of van der Vos et al. [14] which showed areas of apparently lesser activity between the heads of two prostheses, which may interfere with the identification of small structures in the pelvis, such as ovarial cysts or iliac lymph nodes.

Given the potential role of ^18^F-FDG PET for diagnosis of periprosthetic joint infection [16–20], our results may have implications for the clinical interpretation of PET/CT studies for that purpose, but clinical applicability was beyond the scope of the present study and would require a series of patients suspected for periprosthetic infection. A further limitation of our study is that we only studied one type of MAR software. On the other hand, different types of hip implants were included. We chose to exclude movement artefacts between CT and PET, although they are known to enhance overestimation artefacts [3], because they occur only seldom and would obscure the true effect of MAR.

## Conclusions

In a realistic phantom of a hip prosthesis, Smart MAR largely improves quantitative accuracy by recovering counts in underestimated regions. In patient studies, Smart MAR increased SUV in all areas surrounding the prosthesis, but most markedly in the femoral neck region. This increase proves that underestimation of activity is the most prevalent metal artefact. Smart MAR even increases the SUV in the urinary bladder, indicating effects at a distance from the prosthesis. The implications for clinical practice remain to be elucidated in patients suspected of periprosthetic joint infection.

## Data Availability

The datasets used and/or analysed during the current study are available from the corresponding author on reasonable request.

## Abbreviations

AC: Photon attenuation correction
ANOVA: Analysis of variance
CT: Computed tomography
18F-FDG: 2‐[18F]fluoro‐2‐deoxy‐D‐glucose
IQR: Interquartile range
MAR: Metal artefact reduction
OSEM: Ordered subset expectation maximization
PET: Positron emission tomography
ROI: Region of interest
SUV: Standard uptake value
SUV_mean_: Mean standard uptake value
SUV_max_: Maximum standard uptake value
VOI: Volume of interest
